# Antibodies elicited in adults by a primary *Plasmodium falciparum *blood-stage infection recognize different epitopes compared with immune individuals

**DOI:** 10.1186/1475-2875-6-86

**Published:** 2007-07-02

**Authors:** Damon P Eisen, Lina Wang, Helene Jouin, E Elsa H Murhandarwati, Casilda G Black, Odile Mercereau-Puijalon, Ross L Coppel

**Affiliations:** 1 Clinical Centre for Research Excellence in Infectious Diseases, Victorian Infectious Diseases Service, Royal Melbourne Hospital, Grattan St, Parkville, Victoria, 3050, Australia; 2 Malaria and Scabies Unit, Queensland Institute of Medical Research, Herston, Queensland, 4029, Australia; 3 Department of Microbiology, Monash University, Clayton, Victoria, 3800, Australia; 4 Unite d'Immunologie Moléculaire des Parasites, Institut Pasteur, Paris, France

## Abstract

**Background:**

Asexual stage antibody responses following initial *Plasmodium falciparum *infections in previously healthy adults may inform vaccine development, yet these have not been as intensively studied as they have in populations from malaria-endemic areas.

**Methods:**

Serum samples were collected over a six-month period from twenty travellers having returned with falciparum malaria. Fourteen of these were malaria-naïve and six had a past history of one to two episodes of malaria. Antibodies to seven asexual stage *P. falciparum *antigens were measured by ELISA. Invasion inhibitory antibody responses to the 19kDa fragment of merozoite surface protein 1 (MSP1_19_) were determined.

**Results:**

Short-lived antibody responses were found in the majority of the subjects. While MSP1_19 _antibodies were most common, MSP1 block 2 antibodies were significantly less frequent and recognized conserved domains. Antibodies to MSP2 cross-reacted to the dimorphic allelic families and anti-MSP2 isotypes were not IgG3 skewed as shown previously. MSP1_19 _invasion inhibiting antibodies were present in 9/20 patients. A past history of malaria did not influence the frequency of these short-lived, functional antibodies (p = 0.2, 2-tailed Fisher's exact test).

**Conclusion:**

Adults infected with *P. falciparum *for the first time, develop relatively short-lived immune responses that, in the case of MSP1_19_, are functional. Antibodies to the polymorphic antigens studied were particularly directed to allelic family specific, non-repetitive and conserved determinants and were not IgG subclass skewed. These responses are substantially different to those found in malaria immune individuals.

## Background

Understanding the native immune response to *Plasmodium falciparum *infection is an important prerequisite for successful vaccine development against this high priority global disease. Many population studies performed in areas of malaria transmission have correlated the presence of antibodies to *P. falciparum *proteins with protection from disease. In general, the non-sterilizing immunity to malaria seen in adults living in areas hyperendemic for malaria is acquired after numerous infections. Little, however, is known about antibody responses in previously healthy adults after single infections with *P. falciparum*, such as non-immune travellers.

Based on current information, a number of proteins are being advanced as potentially useful components of a subunit malaria vaccine. For example, MSP1_19_, the carboxyl-terminal domain of merozoite surface protein-1 (MSP1) is highly conserved [1], serves a critical function in erythrocyte invasion [2] and has been widely shown to confer protective immunity in various models of malaria infection [3]. The repetitive, block 2 region of MSP1 has been identified as a major immune target by studies that infer immune selection acting upon it and there is a strong association between antibodies to this region and protection [4]. Other proteins, such as MSP2, have been shown in recent field studies of a combination vaccine to induce protective immune responses against homologous strains of malaria [5]. The problem of antigenic diversity can be extreme for some malaria proteins, such as MSP2 and apical membrane antigen-1, and, accordingly, there has been interest in relatively invariant proteins, such as MSP4 [6] and MSP5 [7] that show good degrees of protection against heterologous challenge in model systems of malaria infection [8]. Other conserved proteins, such as MSP6, MSP7 [9] and rhoptry associated membrane antigen (RAMA) [10] induce strong responses during infection and an association has been shown between antibody response and clinical immunity [10,11].

This study has assessed the immune responses in non-immune adults mostly presenting with their first episode of *P. falciparum *infection and characterized the pattern of acquisition of antibodies to seven asexual stage proteins. By systematically collecting a series of samples from these individuals the timing, level and decline of these antibodies has been characterized and a comparison of the immunogenicity of various antigens been made. Antibody responses to overlapping, dimorphic family specific epitopes of MSP1 block 2 and dimorphic family specific repetitive and non-repetitive regions of MSP2 were analysed to determine the specificity of responses to these targets. Immunoglobulin isotyping was performed for antibodies to MSP1_19_, MSP2, MSP4 and MSP7B, where previous data have indicated isotype bias to assess whether these patterns were present. Finally, the functionality of antibodies formed against MSP1_19 _in a growth inhibition assay has been measured.

## Methods

### Blood samples

Full blood and serum samples were collected with written informed consent from twenty patients presenting to the Royal Brisbane and Princess Alexandra Hospitals with falciparum malaria. The Human Research Ethics Committees of the Royal Brisbane and Princess Alexandra Hospitals granted approval for the study. Parasitized erythrocytes were stored in 8M GuHC1. Serum samples were collected at around the time of diagnosis of *P. falciparum *infection then again at approximately one month and six months after infection. Negative controls consisted of 30 samples taken with informed consent from Australian blood donors with no history of exposure to malaria. Positive control sera consisted of samples taken with informed consent from 20 hyperimmune, Papua New Guinean (PNG) adults. The serum samples were stored at -70°C.

### MSP1 block 2 and MSP2 genotyping

A strategy was instituted to detect multiple infections to aid the interpretation of antibody responses to MSP1 block 2 and MSP2 dimorphic family alleles. MSP1 block 2 alleles were amplified and sequenced directly as previously described [12]. MSP2 alleles were amplified by PCR utilising dimorphic family specific oligonucleotides [13]. This approach identified specimens with multiple polymorphic alleles.

### Recombinant proteins

MSP1_19 _(Wellcome strain) was expressed as previously [14]. MSP2 proteins representing; i) the full-length dimorphic family allele (MSP2/3D7 and MSP2/FC27), ii) full length with central repeat region deleted, ie dimorphic family specific non-repetitive region (MSP2/3D7 non-repetitive and MSP2/FC27 non-repetitive) and iii) conserved region alone (MSP2 CR) were expressed as described previously [15]. Full length MSP4 [6], MSP5 [7] and MSP6/MSP7 [9] were all expressed as previously. RAMA-E, the C-terminal erythrocyte-binding domain of RAMA was expressed as previously [10]. All proteins were expressed as GST-fusions, except for MSP4 and MSP6 which were expressed with a C-terminal hexahistidine tag. The conformational fidelity of a number of these proteins cans be confirmed. Reduction and alkylation of the MSP1_19 _recombinant protein has been shown to abolish its reactivity with several inhibitory monoclonal antibodies [14], as well as reduce its recognition by human immune sera [16]. Similar data are available for MSP4 and MSP 5 (Lina Wang, personal communication).

### Antibody measurements

The reactivity of human sera with recombinant proteins and detection of the Ig isotypes of the antigen-specific antibodies, using an isotype-specific method, were examined by ELISA as previously described [17]. Serum samples were diluted 1:500. Positive sera were defined as those that give an absorbance, measured as an optical density (OD) value, greater than the mean plus two standard deviations of absorbance obtained with sera taken from 30 Australian blood donors with no history of exposure to malaria. Positive control sera consisted of pooled samples from 20 hyperimmune, PNG adults. By coating microtitre plates with reference serum and incubating with the human isotype-specific monoclonal antibodies, the absorbances obtained were compared with the actual values for the reference serum and used to calculate compensation factors for the different isotypes, which are the ratios of the absorbance for the given isotype to that of IgG1. The derived compensation factors for IgG1, IgG2, IgG3, and IgG4 were 1, 0.37, 1.07, and 1.71 respectively, and they were used to adjust the ELISA values. Immunoglobulin isotypes were determined on the 20 PNG samples individually to allow comparison with the returned traveller samples.

### ELISA with MSP1 block 2 biotinylated peptides

A set of 82 overlapping 15-mer peptides encompassing all the sequences of the allelic forms of the MSP1 block 2 [1], was synthesized by Chiron Mimotopes Pty. Ltd. (Clayton, Victoria, Australia). The sequences of the peptides are described in a previous study [18]. ELISA with biotinylated peptides was performed as previously described [18]. Each plasma sample was tested in duplicate. The wells that gave a signal two times greater than the OD value of the wells without peptide were considered positive.

### Growth inhibition mediated by MSP1_19 _antibodies

Growth inhibition assays comparing *P. falciparum *(D10 strain) and transfected *P. falciparum *expressing the 19 kDa C-terminal fragment of MSP1 from *P. chabaudi *were performed as previously described [2] to determine the effect of antibodies specific for MSP1_19 _on parasite growth. In brief, mature stages from synchronized parasite cultures were incubated with patient and control, pooled non-immune human serum diluted to 1:10, for 26 hours to allow for schizont rupture and merozoite invasion. Invasion inhibition for each parasite line was assessed microscopically by comparing parasitaemia observed in the presence of test and non-immune serum.

### Statistical analysis

The frequency of positive antibody responses was compared using the Chi-squared test. Comparisons between non-parametric variables such as median ODs and immunoglobulin isotype ODs were performed using the Mann-Whitney test. Correlations between independent variables were performed using Pearson's coefficient. Invasion inhibition due to MSP1_19_antibodies was determined by comparing mean invasion rates for the two parasite lines using a two-sample Student's *t *test. All statistical analyses were carried out in Minitab^® ^Release 14.

## Results

### Patients

Twenty patients participated in the study. There were ten male and ten female patients. The median age was 34 years. Twelve patients acquired *P. falciparum *in the Western Pacific region (PNG or the Solomon Islands), six in Africa and two in Asia. There were no mixed infections. The median parasitaemia was 24,900/μL (740-326,000/μL). Fourteen of the patients had not had *P. falciparum *previously. These patients were returning travellers from malaria endemic areas. One other returned traveller had two earlier episodes of *P. falciparum *in the six months before the study episode. Five patients were born in malaria endemic areas and had a past history of malaria occurring between approximately 20 years and 3 months before this study. These six patients had one or two previous episodes of malaria.

Fifty-five samples were collected from the 20 returned travellers. At around the time of diagnosis (0–2 weeks) 21 samples were collected. At approximately one month post diagnosis (4–8 weeks) 11 samples were collected and at up to six months post diagnosis (10–41 weeks) 23 samples were collected. Multiple samples were available from some travellers at some of these time points.

### Frequency of positive antibody responses (Table 1 and 2)

**Table 1:** Rates of positive antibody responses measured by ELISA to merozoite surface proteins

	Proportion +ve serology
MSP1_19_	19/20
FC27 non-repetitive	18/20
FC27/3D7 non-repetitive	17/20
3D7	16/20
MSP2 conserved region/MSP6/RAMA-E	15/20
MSP4/MSP5	14/20^a^
MSP7	13/20^a^
MSP1 block 2	4/20^b^

^a ^proportion +ves < MSP1_19_, p < 0.05

^b ^proportion +ves < MSP1_19_, p < 0.0001

**Table 2:** Antibody responses compared with negative control sera and in relation to time since infection, past infection and parasitaemia

Median optical density	MSP1_19_	MSP2 3D7	MSP2 FC27	MSP2 3D7 non-repetitive	MSP2 FC27 non-repetitive	MSP2 conserved region	MSP4	MSP5	MSP6B	MSP7B	RAMA-E
Overall (n = 55)	1.273^a^	0.296^a^	0.285^a^	0.939^a^	0.456^a^	0.291^a^	0.578^a^	0.35^a^	0.099^a^	0.295^a^	0.091^a^
0–2 weeks (n = 21)	2.95^a^	0.421^a^	0.681^a^	1.095^a^	0.635^a^	0.362^ac^	0.885^a^	0.407^a^	0.141^ac^	0.582^a^	0.089^a^
4–8 weeks (n = 11)	1.65^ad^	0.538^a^	0.511^a^	1.841^ad^	0.597^ad^	0.344^ad^	0.694^a^	0.412^ad^	0.154^ad^	0.295^a^	0.192^ad^
10–41 weeks (n = 23)	0.46^a^	0.137^a^	0.151^a^	0.497^a^	0.23^a^	0.146^a^	0.415^a^	0.292^a^	0.047^a^	0.25^a^	0.057^a^
No past infection (n = 37)	0.681^ae^	0.157^a^	0.229^a^	0.849^a^	0.413^a^	0.32^a^	0.61^a^	0.24^af^	0.068^a^	0.284^a^	0.089^a^
Past infection (n = 18)	2.82^a^	0.975^a^	0.748^a^	1.098^a^	0.598^a^	0.274^a^	0.463^a^	0.402^a^	0.121^a^	0.404^a^	0.114^a^
Lower quartile parasitaemia (n = 14)	0.252^a^	0.101^a^	0.08^b^	0.656^a^	0.18^bg^	0.221^b^	0.419^b^	0.335^b^	0.043^b^	0.285^b^	0.075^b^
Upper quartile parasitaemia (n = 14)	0.83^a^	0.157^a^	0.428^a^	0.684^a^	0.645^a^	0.525^a^	0.708^b^	0.198^b^	0.069^a^	0.176^a^	0.071^a^
Negative control (n = 30)	-.004	0.009	0.015	0.061	0.044	0.069	0.086	0.074	0.010	0.055	0.013

^a ^differs from neg controls p < 0.0001

^b ^differs from neg controls p < 0.05

^c ^Timepoint 1 vs timepoint 3 median difference p < 0.05

^d ^Timepoint 2 vs timepoint 3 median difference p < 0.05

^e ^No past infection vs past infection median difference p < 0.05

^f ^No past infection vs past infection median difference p < 0.01

^g ^Lower vs upper quartile parasitaemia median difference p < 0.05

Antibody responses in patient sera were measured against the seven asexual stage antigens (MSP1, MSP2, MSP4, MSP5, MSP6/7, and RAMA-E). Positive antibody responses as defined above were counted in any of the samples from a study patient. The highest frequency of positive antibody responses to the blood stage antigens tested was to MSP1_19 _(19/20). Importantly, 15/20 patients had antibodies to the conserved region of MSP2. A significantly lower frequency of positive antibody responses compared with MSP1_19 _antibodies were seen for MSP4/MSP5 (14/20), MSP7 (13/20) and MSP1 block 2 antibodies (4/20) (Table 1). Median antibody levels from *P. falciparum *cases were significantly higher than median negative control antibodies for all antigens tested (Table 2).

### Time course of antibodies, influence of past infection and parasitaemia (Table 2)

Median absorbances were significantly greater than negative control results at the first time point at around 0–2 weeks after infection. The general trend in absorbances was for levels to be unchanged at 4–8 weeks and to decline significantly after 10 weeks. At the last time point measured, the median absorbances for all antigens tested remained higher than negative controls. Samples were grouped according to whether patients had a history of previous malaria or not. Median absorbances were significantly higher in patients with past malaria infection only for anti-MSP1_19 _and anti-MSP5 responses. Otherwise, for all other antigens tested, the median absorbances did not differ according to whether patients had a previous history of malaria or not. Peripheral blood parasite counts appeared to have little impact on the antibody response for most antigens tested. The only antibody response where there was a significant difference in the median ODs between patients in the upper (67,000/μL) and lower (14,484/μL) parasitaemia quartiles was against MSP2/FC27 non-repetitive regions. There was no significant correlation between parasite counts and antibody levels for any antigen.

### MSP2 antibody responses (Table 3)

**Table 3:** Correlation of antibody responses to MSP2 recombinant antigens expressing allelic family specific and conserved regions; a, antibodies in all patients b, antibodies in naïve patients

			Pearson's correlation coefficients (r)	
	3D7 non-repetitive	FC27	FC27 non-repetitive	MSP2 conserved region
All infections (n = 20, 2 non-typeable)				
3D7	0.96^a^	0.51^b^	0.42	0.55^b^
3D7 non-repetitive		0.48^b^	0.4	0.67^a^
FC27			0.85^a^	0.13
FC27 non-repetitive				0.27
				
All single allelic family infections (n = 15)				
3D7	0.94^a^	0.55^b^	0.18	0.33
3D7 non-repetitive		0.48	0.17	0.51
FC27			0.83^a^	0.13
FC27 non-non-repetitive				0.17
				
Single 3D7 infections (n = 11)				
3D7	0.95^a^	0.89^a^	0.78^b^	0.6
3D7 non-repetitive		0.82^b^	0.85^a^	0.72^b^
FC27			0.57	0.43
FC27 non-repetitive				0.92^a^
				
Single FC27 infections (n = 4)				
3D7	0.96^b^	0.33	-0.14	-0.35
3D7 non-repetitive		0.39	-0.12	-0.24
FC27			0.87	-0.81
FC27 non-repetitive				-0.78
				
Multiple 3D7/FC27 infections (n = 3)				
3D7	0.98	0.79	0.89	0.63
3D7 non-repetitive		0.67	0.79	0.76
FC27			0.98	0.03
FC27 non-repetitive	0.21			

^a ^p < 0.001

^b ^p < 0.05

Responses to recombinant MSP2 antigens containing various family specific and conserved regions of the molecule in the first patient sample collected were correlated to assess for cross-reactive specificities. Patient samples from *P. falciparum *infections containing multiple MSP2 family alleles were identified by the MSP2 dimorphic family specific PCR. Three patient samples had multiple 3D7 and FC27 family alleles, 11 had single 3D7 alleles and four had single FC27 alleles. In two samples, MSP2 alleles could not be determined. Cross-allelic family reactive antibodies were found in this study, as there were significant correlations between the responses to 3D7 and FC27 recombinant antigens. Firstly, correlations of responses in all patients, malaria-naïve or previously exposed, were analysed. (Table 3a) Significant correlations were found in the entire sample set (ie. single 3D7 or FC27 infections, multiple 3D7/FC27 allelic and untypeable infections) but were also present when sera from patients shown to have single infections with 3D7 alleles alone were analysed. Significant correlations were present between antibodies to the conserved region of MSP2 and the dimorphic family specific non-repetitive MSP2 allelic and, to a lesser extent, full length MSP2 family constructs. The correlation between MSP2 conserved region and dimorphic family specific non-repetitive MSP2 allelic family constructs was most pronounced when the sera from 3D7 infections alone were analysed. When the samples from malaria-naïve patients alone were analysed (Table 3b), cross-allelic family reactions were also present, but to a lesser extent. These are shown by the significant correlations present between the FC27 non-repetitive construct and the conserved region in 3D7 allelic family infected individuals and between 3D7 recombinant antigens in FC27 allelic family infected individuals.

**Table 3b:** Naïve patients

			Pearson's correlation coefficients (r)	
	3D7 non-repetitive	FC27	FC27 non-repetitive	MSP2 conserved region
All infections (n = 14, 2 non-typeable)				
3D7	0.94^a^	0.25	0.24	0.5
3D7 non-repetitive		0.21	0.24	0.64^b^
FC27			0.92^a^	-0.17
FC27 non-repetitive				0.21
				
All single allelic family infections (n = 10)				
3D7	0.85^b^	0.45	0.17	-0.12
3D7 non-repetitive		0.31	0.11	0.12
FC27			0.93^a^	-0.11
FC27 non-non-repetitive				0.03
				
Single 3D7 infections (n = 6)				
3D7	0.81	0.74	0.36	0.03
3D7 non-repetitive		0.45	0.63	0.46
FC27			0.32	0.09
FC27 non-repetitive				0.89^b^
				
Single FC27 infections (n = 4)				
3D7	0.96^b^	0.33	-0.14	-0.35
3D7 non-repetitive		0.39	-0.12	-0.24
FC27			0.87	-0.81
FC27 non-repetitive				-0.78

^a ^p < 0.001

^b ^p < 0.05

### Immunoglobulin isotypes (Table 4)

**Table 4:** Comparison of median optical densities of immunoglobulin isotypes between study samples and PNG, positive control sera.

	MSP1_19_	MSP2 3D7	MSP2 FC27	MSP4	MSP7B
IgG1	NS^a^	NS	NS	NS	0.016 : 0.044^b^
IgG2	NS	NS	0.005 : 0.024^b^	0.038 : 0.017^c^	NS
IgG3	NS	0.009 : 0.09^b^	0.003 : 0.35^b^	NS	0.001 : 0.155^c^
IgG4	NS	NS	NS	NS	-0.002 : 0.006^c^
IgM	NS	NS	NS	NS	NS

^a ^not significantly different

^b ^p < 0.05

^c ^p < 0.001

The isotypes of anti-merozoite surface protein antibodies were compared among sera from study subjects and sera from long-term residents of PNG who had experienced multiple malaria infections. Significantly lower median titres were seen in IgG2 responses to the MSP2 FC27 allele and MSP4 in study patients compared to PNG residents. Similarly, IgG3 responses to the MSP2 3D7 and FC27 allelic families and MSP7 were lower than in PNG controls. Correlations between IgG1 and IgG3 responses were compared in study and PNG control sera. In study and control sera, there was a strong IgG1 predominance for MSP1_19_. The expected bias to IgG3 for anti-MSP2 and anti-MSP4 responses was not present in the study sera, but instead there was IgG1 predominance, although less marked than for MSP1_19_. Comparison of IgM responses in study patients who were naïve and those who had a previous history of malaria showed no significant differences for all antibodies tested.

### MSP1 Block 2 antibody responses

Antibodies to the MSP1 block 2 epitopes were found in a minority of patients (4/20). In these patients, antibodies were found to be restricted to one allelic family type in three of the four patients and were also restricted to a maximum of four epitopes. The same temporal pattern as with the other antigens was found with relatively high absorbances at the time of infection followed by a reduction over the next six months. In one patient, antibodies to a single K1 and RO33 family allele could be detected. This individual had no past history of malaria infection. Genotyping of the MSP1 block 2 PCR showed the presence of a single K1 dimorphic family allele in this individual.

### Growth inhibition due to MSP1_19 _antibodies

Significant invasion inhibiting anti-MSP1_19 _antibodies were detected in a total of nine patient samples. In these samples, there was a 14.8 – 48.7% reduction of red blood cell invasion when *P. falciparum *was compared with the *P. falciparum *MSP1_19 _– *P. chabaudi *transfectant. The antibodies were short-lived, being present soon after the patent infection (median 36 days; range 9–249 days) and declining in 5/9 cases (tested negative at median 185 days post infection; range 170 – 236 days.). In three cases, the invasion inhibiting antibodies were detected using the standard assay conditions and serum dilution of 1:10. However, nine serum samples caused inhibition of schizont rupture, which interfered with assessment of growth inhibition at the standard dilution. When these sera were diluted to 1:90, six were found to contain invasion inhibiting anti-MSP1_19 _antibodies invasion. There was no difference in frequency of these functional MSP1_19 _antibodies according to whether patients had a past history of malaria or not (p = 0.2, 2-tailed Fisher's exact) but anti-MSP1_19 _absorbances were significantly higher in the group with invasion inhibiting antibodies (2.31 vs. 0.49 p < 0.05).

## Discussion

Antibody responses to malaria in people living in endemic regions are complex and represent the summation of multiple infections, typically beginning in infancy. Repeated infections are required to stimulate the non-sterile immunity found in adults living in malaria hyperendemic areas. Little is known about the immune responses to a single *P. falciparum *infection. In the group of previously healthy, mostly *P. falciparum *naïve, adults studied here, antibodies were measured to seven different malaria proteins including multiple MSP1 and MSP2 regions and alleles. Relatively short-lived antibody responses were found most commonly with MSP4/5/7 and MSP1 block 2 antibodies being significantly less frequent than the rest of the antibodies tested. In nine subjects, functional, invasion inhibiting MSP1_19 _antibodies were shown to be present. Importantly, frequent antibody reactions to conserved regions of MSP2 have been documented for the first time in humans exposed to a single *P. falciparum *infection. Similarly, the MSP2 isotype response was not skewed towards IgG3 antibodies as previously shown [19]. These features of the immune response to *P. falciparum *seen in naïve adults contrast markedly with the MSP2 repeat region dominated response found in malaria endemic populations [19].

MSP1_19 _appeared to be particularly immunogenic in this population as shown by the presence of invasion inhibiting antibodies in 9/20 patients that were present one month after infection but mostly disappeared by six months. Of interest, a significant proportion (45%) of sera inhibited schizont rupture in growth assays, a property much more rarely encountered in sera from endemic areas (data not shown). Dilution of these sera revealed an underlying capacity to inhibit merozoite invasion. Functional MSP1_19 _antibodies, as detected by this method, were correlated with protection from *P. falciparum *infection in Kenyan children and adults while MSP1_19 _antibodies measured by ELISA were not [20]. Other studies do not show the same level of correlation and the exact protective importance of this antibody subpopulation is not yet defined in all populations [21,22]. Nevertheless, it seems reasonable to suggest that the presence of such antibodies indicates a capacity of convalescent sera to exert a level of anti-parasitic action after a single infection. It will be particularly interesting to discover whether similar levels of inhibitory antibodies are induced in children resident in endemic areas, after their first bout of malaria. This may represent a previously unrecognized mechanism for short-term host protection.

MSP1 block 2, by contrast, was much less antigenic. Only 4/20 of the adult population were positive for any MSP1 block 2 antibodies. This contrasts with previous field studies that also measured MSP1 block 2 antibodies to allelic family specific, short peptides. An age-dependant increase in antibodies to 58.3% of 10–15 year old Ghanaian children was found [23]. The low frequency of antibodies MSP1 block 2 in this study does not appear to be due to failure to detect short-lived antibodies [24] as the patients were assayed immediately after their clinical episode of malaria with fine epitope mapping using 15 mer peptides that cover the MSP1 block 2 locus with significant redundancy. The epitopes recognized in this study were all from relatively constant regions of the MSP1 block 2 alleles. One patient who had no past history of malaria infection had antibodies detected to both K1 and RO33 allelic family antigens despite being infected with only a K1 family isolate. The observed low antigenicity of MSP1 block 2 in adults mostly after their first *P. falciparum *infection suggests that the potentially protective antibodies to this region [23] reflect a high frequency of previous episodes of malaria. The other antigens assayed with relatively low levels of seroreactivity were MSP4/5 and MSP7, which, like MSP1 block 2, appear to have higher rates of seropositivity in malaria endemic populations [9,17].

For the first time, in this study, there is evidence of commonly occurring antibody responses to the conserved regions of MSP2. This contrasts with prevailing evidence that suggests the immunodominance of the central amino acid repeat region following repeated infections of humans [19] or immunization studies [25]. Antibody responses to the conserved regions of MSP2 were documented in 15/20 of study patients. By contrast, antibodies from pooled PNG adult serum to this region were below the cut-off level so this result is highly significant. When the same methodology was used to study semi-immune Vietnamese teenagers and adults, only 1/15 had antibodies to the MSP2 conserved region [15]. No measurable antibodies to the conserved regions of MSP2 were induced by vaccination with the 3D7 allele in five to nine year old children in the Combination B trial [25]. A minority of non-immune Swiss travellers have been shown to have antibodies to the conserved regions of MSP2 by non-quantitative immunoblot [26].

Additionally, significant correlations between MSP2 allelic family constructs have been shown. More significant correlations between antibodies were found in individuals with a past history of one to two episodes of malaria. Even in previously naïve individuals, apparently cross-allelic family reactive antibodies were present. It appears that these are attributable to antibodies to the conserved region of MSP2. The correlations we have calculated are based on small numbers of samples. Previous studies of anti-MSP2 immune responses in populations living in hyperendemic areas have shown that antibodies are dimorphic family specific [19], but that there is extensive intra-dimorphic family immune response cross-reactivity [27]. Vaccine-derived MSP2 antibodies were restricted to the 3D7 specific regions of the vaccine antigen [25]. Limited inter-dimorphic family antibody cross reactivity has been recognized previously for MSP2 after patent malaria in two non-immune patients [26] and in this study, inter allelic family specific antibodies to MSP1 block 2 were found in one patient.

Protective antibodies to repeat regions of MSP2 have been shown to change from IgG1 to IgG3 dominated responses with aging indicating a maturation of the immune response [28,29]. The patients studied here do not show this same isotype pattern and MSP2 antibodies were predominantly IgG1. Thus, these non-immune adults studied herein have MSP2 responses that differ in target and isotype from hyperimmune sera. It may be that the repeat region dominated MSP2 responses seen in malaria endemic regions result from the repeated infections experienced by infants with immature immune systems that do not recognise the conserved regions of MSP2. Similarly, the IgG3 dominated anti-MSP7 response in immune adults [9] was not seen in this study population. Previously naïve patients were also not found to have higher IgM responses to any of the antigens tested than adults with a past history of malaria.

Antibody responses trended to declining levels over the 6 months of follow up. At the final bleed though, antibodies to all antigens tested remained positive. The level of parasitaemia, not unexpectedly, had essentially no influence on the level of antibodies measured. In field settings with seasonal malaria transmission, MSP1_19 _and MSP1 block 2 antibodies have been shown to decrease over dry seasons [24]. Antibodies to chondroitin sulphate A-binding variant surface antigens have also been shown to be short-lived in women with pregnancy associated malaria but their presence correlates with protection against infection of the placenta [30]. Temporary humoral immune responses seem to be common for malaria antigens.

## Conclusion

Viewed together, the antibody responses measured to MSP1 block 2 and MSP2 suggest that singly infected adults have a very different immune response in comparison with those living in malaria-endemic areas. It appears that antibodies to MSP1 block 2 become much more common with repeated infection while those to MSP2 become dominated by allelic family specificities particularly to the central amino acid repeat region with extinction of responses to the conserved region. The development of cross allelic-family reactive MSP2 antibodies after a single native infection with *P. falciparum *has been documented. These data strongly support the use of MSP2 for malaria vaccination. These data are also supportive of MSP1_19 _vaccine development given it was highly immunogenic after single infection with functional antibodies measured. As functional MSP1_19 _antibodies have been shown to be correlated with protection [20] it now appears to be imperative to develop assays to test for functional antibodies in future vaccine trials of other antigens, such as MSP2.

## Authors' contributions

DE conceived of the study, was responsible for its design and coordination, performed the statistical analysis and drafted the manuscript. LW carried out the antibody assays and reviewed the manuscript. HJ carried out the MSP1 block 2 antibody assays. EM and CB carried out the MSP1_19 _invasion inhibition assays. OMP participated in the design of the study and contributed to the drafting of the manuscript. RC conceived of the study, and participated in its design and coordination and helped to draft the manuscript. All authors read and approved the final manuscript.
